# Seasonal Dynamics of Algal Communities and Key Environmental Drivers in the Subpolar Front Zone off Eastern Korea

**DOI:** 10.3390/biology14070738

**Published:** 2025-06-20

**Authors:** Pyo Il Han, Hyun Soo Rho, Joo Myun Park, Beom-Sik Kim, Jong Won Park, Dongyoung Kim, Dong Young Lee, Chung Il Lee

**Affiliations:** 1 Department of Marine Ecology and Environment, Gangneung-Wonju National University, Gangneung-si 25457, Republic of Korea; hanpoyil200@naver.com (P.I.H.); 20131078@gwnu.ac.kr (B.-S.K.); po7639@gwnu.ac.kr (J.W.P.); dongyoung@gwnu.ac.kr (D.K.); perfectiony@gwnu.ac.kr (D.Y.L.); 2 East Sea Research Institute, Korea Institute of Ocean Science and Technology, Uljin-gun 36315, Republic of Korea; hsrho@kiost.ac.kr; 3 Department of Marine Biology, Kunsan National University, Gunsan 54150, Republic of Korea; joomyun@kunsan.ac.kr

**Keywords:** algal community structure, seasonal change, environmental factor, subpolar front zone, eastern coast of Korea

## Abstract

Located within the subpolar front (SPF) zone, the eastern coast of Korea is subject to frequent environmental changes on both spatial and temporal scales. In this study, we analyzed seasonal changes in the algal community structure and related environmental factors at two sites, Chodo (CD) and Sageunjin (SG), which were influenced by cold and warm-water masses, respectively, in the SPF zone. Sea surface temperature (SST) exhibited greater seasonal change in CD, whereas significant wave height was higher in SG. The salinity and nutrient concentrations decreased during summer and increased in winter at both sites. Seasonal shifts in the dominant species were distinct, with turf species positively correlated with SST dominating the intertidal zone of SG during winter, and canopy species were associated with nutrient concentrations dominating the intertidal zone of CD. Bryopsidales, which was positively correlated with the N:P ratio, dominated the SG subtidal zone in summer, whereas Fucales, which was positively correlated with the SST, dominated the CD subtidal zone. These results enhance our understanding of seaweed responses to environmental changes in SPF zones and provide a basis for predicting future changes in coastal ecosystems.

## Simple Summary

In this study, we investigated key environmental factors influencing seasonal changes in algal communities within the subpolar front zone. Water temperature was the main driver of community shifts in the north and was composed of species with varying thermal tolerances. In contrast, nutrient concentrations shaped the southern community, which was dominated by eurythermal species. These results indicated that the environmental factors affecting algal community structure may vary across sites and suggested that the environmental characteristics of seaweeds influence algal community structure. Overall, these results enhance our understanding of how marine environments influence changes in community structure.

## 1. Introduction

Seaweeds are primary producers in coastal ecosystems, supplying food for benthic organisms [1,2] and spawning grounds for pelagic species [3]. As sessile organisms, seaweeds are highly sensitive to environmental and algal community structure changes in response to environmental factors, particularly in terms of species composition and biomass [4,5]. A case in point is the shift in dominant species from Laminariales, which favors colder waters, to Fucales, which thrives in warmer conditions in response to warming sea surface temperature (SST) on the western coast of Australia [6]. Environmental sensitivity can alter key physiological processes in seaweeds, such as survival, growth, and reproduction. Moreover, environmental factors, such as water temperature, salinity, nutrient concentration, and wave action, vary across both temporal and spatial scales, and community structure changes in response to habitat conditions [7,8]. The biomass of nutrient-efficient species typically increases in eutrophic waters, characterized by a high attachment capacity, and often dominates in areas with strong wave action [9,10].

In summer, warming water temperatures and decreasing salinity owing to increased freshwater input promote the dominance of eurythermal and euryhaline species. Conversely, lower water temperatures and higher salinity in winter lead to the reappearance of previously dominant species, such as Laminariales, accompanied by a decline in the biomass of summer-dominant taxa [11,12]. Community structure can vary in response to regional and seasonal environmental changes, and its sensitivity to these changes makes it a useful bioindicator for indirectly assessing the environmental status of coastal ecosystems [5]. Therefore, understanding how community structure responds to seasonal environmental changes is important for assessing the status of coastal ecosystems and developing effective fisheries resource management strategies to respond to coastal environmental changes.

Environmental conditions across climatic zones constrain algal physiological performance and drive changes in community structure among zones [13,14,15]. Tropical Bryopsidales dominate the tropical Indian Ocean [16], whereas temperate species such as Laminariales and Fucales dominate the temperate oceans of the Northeast Pacific and southeastern Australia [17,18]. However, community structure differs between sites, even within the same climate zone, and is influenced by differences in salinity, nutrient concentrations, and wave heights [9,10]. In the eastern Atlantic Ocean along the French coast, Laminariales dominate areas with relatively high wave heights, whereas Fucales tend to be dominant in areas with lower wave height [9].

A frontal zone is a boundary where water masses with differing characteristics converge and is typically associated with frequent environmental fluctuations resulting from changes in water mass distribution. Seaweeds in the frontal zones are frequently exposed to environmental changes that affect species composition and biomass. The eastern coast of Korea (ECK), where the East Korea Warm Current intersects with the North Korea Cold Current, is part of the subpolar front (SPF), which extends from 36.4° N to 40.1° N [19,20,21,22,23,24]. The location of the SPF shifts in response to changes in water mass distribution and inflow of warm currents into the East (Japan) Sea [23]. Its intensity increases from autumn to early winter (November to January) [22,23]. Seasonal dynamics in the front distribution within SPF zones can rapidly alter algal communities, including species composition and biomass [9,10,25].

Global warming has driven both the extinction of native species and the invasion of non-native species, resulting in rapid shifts in community structure [26,27]. In the ECK, ocean warming has expanded subtropical Bryopsidales northward and reduced boreal Laminariales, potentially threatening the sustainability of coastal ecosystems and fisheries [28,29].

Previous studies on community structure have primarily examined broad geographic scales, focusing on the patterns of community change in response to water temperature. However, studies on seasonal changes in communities within frontal zones where frequent environmental fluctuations remain limited, particularly those considering multiple environmental factors. Therefore, the aim of this study was to understand the characteristics of changes in the algal community structure and to understand the relationship between community dynamics and key environmental drivers at two distinct sites in the SPF zone along the ECK. We hypothesized that the seasonal community composition in the SPF zone varies with the influence of different water masses. Sites more affected by cold waters support a mix of species with distinct environmental preferences, whereas sites influenced by warm waters are dominated by species with broader environmental tolerance. We examined the seasonal shifts in community structure and environmental variables at two sites in the SPF zone that differed in seasonal dynamics to test this hypothesis.

## 2. Materials and Methods

### 2.1. Study Site

**Figure 1:**
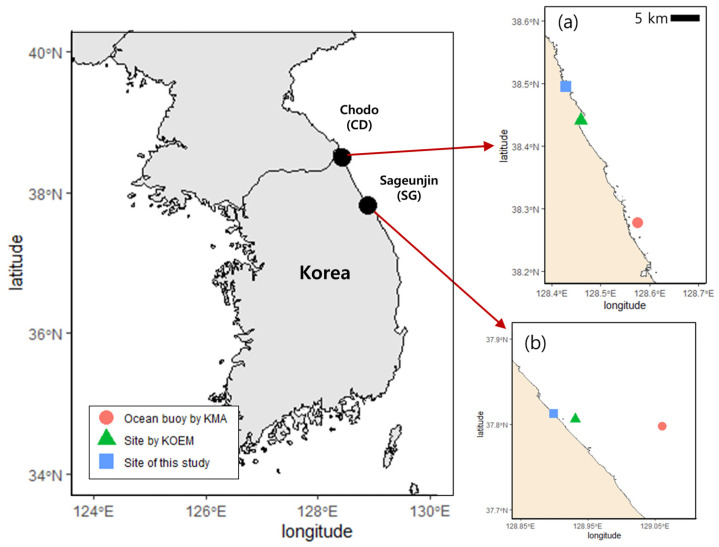
Location of field survey and long-term observation stations. (**a**) Chodo (CD); (**b**) Sageunjin (SG); scale bar: 5 km; ocean buoy data were obtained from the Korea Meteorological Administration (KMA); nutrient data were provided by the Korea Marine Environment Management Corporation (KOEM).

The SPF extends from 36.4° N to 40.1° N off the ECK, encompassing the study sites [22,24]. Field surveys were conducted in the intertidal and subtidal zones in the SPF zone, separating the area relatively more affected by cold-water mass in Chodo (CD, 38°29′40.8″ N, 128°25′47.1″ E) from that more affected by warm-water mass in Sageunjin (SG, 37°48′46.5″ N, 128°53′58.0″ E). The two survey sites are approximately 90 km apart and share similar topographic characteristics; they are open and unprotected from incoming waves from the open ocean. Fieldwork was conducted four times a year for each season from February 2023 to November 2024, for a total of eight times (Figure 1).

### 2.2. Marine Environment

Marine environmental surveys were conducted using field surveys and long-term observations of water temperature, salinity, nutrient concentrations, and wave height to identify the key environmental factors that may influence changes in community structure.

The water temperature, salinity, and nutrient concentrations were measured at each survey station during the day. Water temperature and salinity were recorded every 5 s using a CT sensor (Duo-3, RBR Ltd., Kanata, ON, Canada) at a depth of 0.5–1.0 m. Seawater samples for nutrient measurements were collected from surface waters using sterile 6 L bottles at the same sites where water temperature and salinity were measured and were transported to the laboratory within 6 h. The transported samples were subsequently frozen at −4 °C for storage and stabilized at ambient temperature within half a day prior to measurements. The samples were filtered using a syringe filter (pore size: 0.45 µm) in the laboratory prior to measurements. Nutrient concentrations were measured using a spectrophotometer (LIBRA, Biochrom, Cambridge, UK) by measuring the absorbance of the colored solutions using the indophenol method for ammonium (NH_4_^+^), the diazotization method for nitrite (NO_2_^−^), the cadmium–copper column method for nitrate (NO_3_^−^), and the ascorbic acid method for phosphate (PO_4_^3−^) by the spectrophotometer method [30].

Long-term observational data were obtained from stations near the field survey sites, which provided continuous records. Water temperature and significant wave height were obtained from wave buoys close to CD (38°16′38.3″ N, 128°34′32.5″ E) and SG (37°47′53.9″ N, 129°3′38.9″ E), operated by the Korea Meteorological Administration (KMA). Ten-year monthly means were calculated based on hourly buoy data collected between January 2014 and December 2023. Salinity and nutrient concentrations were obtained from seasonal offshore observations (February, May, August, and November) conducted by the Korea Marine Environment Management Corporation (KOEM) close to CD (38°26′25.0″ N, 128°27′35.0″ E) and SG (37°48′23.0″ N, 128°55′54.0″ E). These data, including salinity, DIN, and DIP, were collected as part of the Marine Environmental Measurement Network (https://meis.go.kr/mei/observe/port.do, accessed on 22 March 2025) during the same period as wave buoy deployment (Figure 1).

### 2.3. Algal Community Structure

The algal community was seasonally surveyed in an area with a tidal range of no more than 30 cm throughout the year [31]. Because of minimal tidal differences, the survey site was divided into intertidal and subtidal zones at a depth of 1 m. Six quadrats (three per zone) were placed in the intertidal and subtidal zones during each survey to collect seaweed. Seaweed samples were obtained by placing a 0.5 m × 0.5 m quadrat on the rock surface where algae were attached and collecting all specimens within the quadrat. Species identification was determined on their morphological characteristics (e.g., thallus shape, growth size, branch shape, number of cell layers, holdfast shape, etc.) using the National Inventory of Biological Species (www.kbr.go.kr, accessed on 5 March 2025), the literature [32,33,34,35,36,37,38,39,40] provided by the National Institute of Biological Resources (www.nibr.go.kr, accessed on 5 March 2025), and the international database AlgaeBase (www.algaebase.org, accessed on 8 April 2025). Tissue sections of seaweeds and small species were observed using a light microscope (IX70, Olympus, Tokyo, Japan) for identification. Each species was weighed to the nearest 0.01 g using an electronic scale, and biomass was calculated as wet weight per unit area. Algae were categorized at the Phylum and Order levels based on taxonomy [41]. The number of Orders categorized as subdivisions of Phylum was 3 in the Chlorophyta, 7 in the Ochrophyta, and 11 in the Rhodophyta, totaling 21 Orders across 3 Phyla, along with Melobesioidean algae. The functional groups of the algae were categorized into canopy, subcanopy, and turf species by referring to a previously described method [42] (Table 1).

### 2.4. Data Analysis

Time series of monthly mean SST and significant wave height were analyzed, and seasonal mean salinity and nutrient concentrations over the same period were compared to assess the seasonal environmental variability among the survey sites.

We compared the abundance and biomass of species by taxonomic classification by season and the relative biomass of each taxonomic class to the total biomass to determine seasonal changes in the algal community structure. Taxa with very small individual weights or very few individuals, representing ≤2% of the seasonal mean in their composition, were unsuitable for explaining seasonal change and were combined and labeled as Other. In addition, encrusting species (e.g., Ralfsiales and Melobesioidean algae) in the functional group were excluded from comparisons of relative biomass percentages because they were found attached to the surface of rocks or the thallus of other algae, limiting quantitative sampling, and were only recorded based on their presence or absence. Each taxon was divided into ecological status group (ESG) I and ESG II species based on morphological characteristics, as previously described [5,45], to analyze the ecological index, which can indirectly evaluate the environmental conditions, reflected in the principal component analysis (PCA) component. Ecological indices, ecological evaluation index (EEI-c), and ecological quality ratio (EQR) were calculated from the proportion of late succession ESG I species, early succession ESG II species, and species richness as previously described based on the methods of [5,45,46], respectively, to represent the state of the algal community by season [47,48]. Species richness (Margalef’s index) and the Shannon diversity index were calculated based on the number of species observed in each season [49]. Spatial and temporal similarities were then evaluated using Bray–Curtis similarity based on the species richness values. PCA was used to investigate the comprehensive relationship between environmental factors and algal community composition within the SPF zone. All variables in the environmental and biological data were analyzed using R software (version 4.4.0). The principal components explaining the main variability were extracted based on the standardized data matrix, and each principal component (PC) was sorted according to its contribution to the variance and used for interpretation. The loading values were then calculated using the “factoextra” package to identify the main environmental factors [50,51].

## 3. Results

### 3.1. Seasonal Changes in Environmental Factors

**Figure 2:**
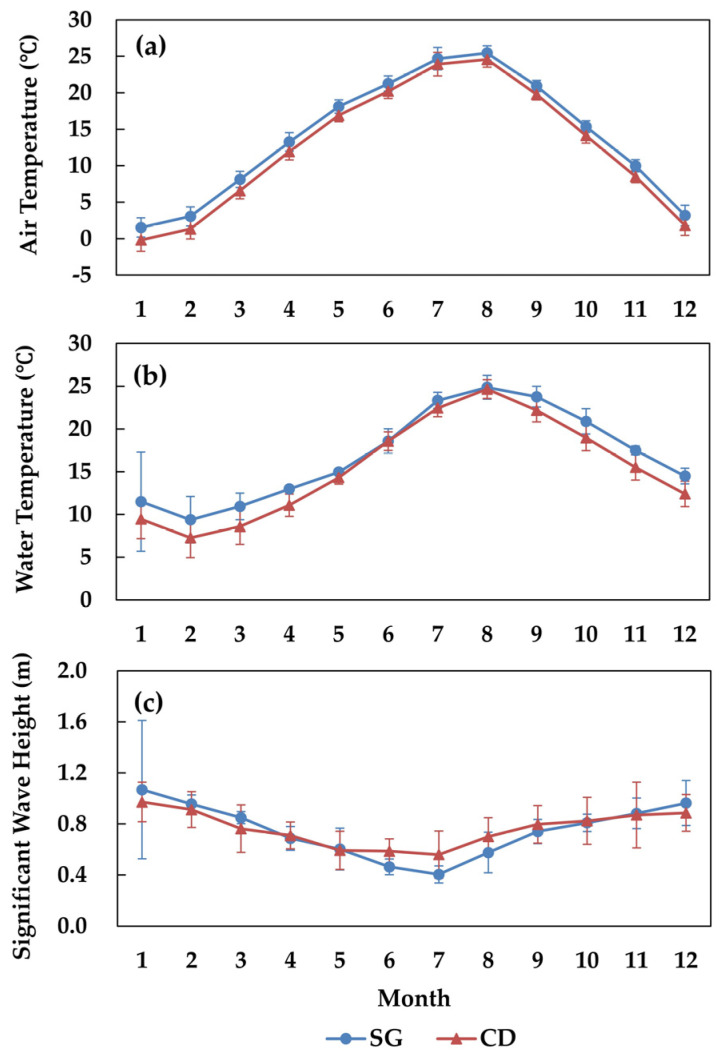
Ten-year (2014–2023) monthly mean data for Sageunjin (SG) and Chodo (CD). (**a**) Air temperature; (**b**) water temperature; (**c**) significant wave height.

**Figure 3:**
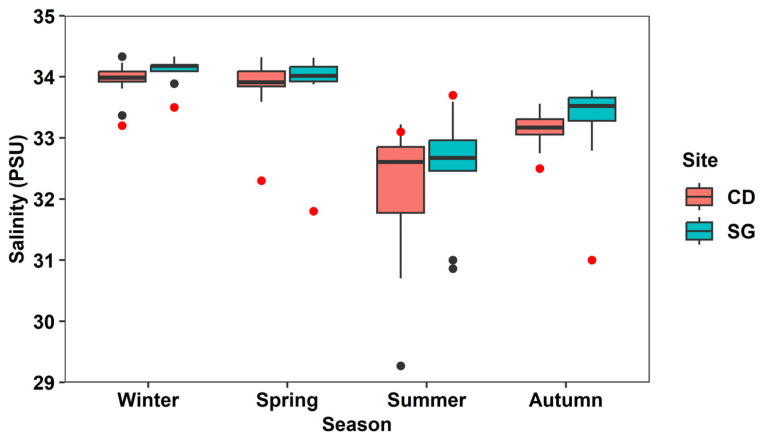
Ten-year (2014–2023) monthly mean salinity concentrations in Sageunjin (SG) and Chodo (CD). Black dots: outlier data; red dots: field survey data; source: Korea Marine Environment Management Corporation (KOEM).

In SG, the monthly mean water temperature ranged from 1.6 ± 0.2 °C in January to 26.4 ± 1.6 °C in August, while in CD it ranged from 0.1 ± 0.4 °C in January to 25.7 ± 0.5 °C in July (Figure 2a). The monthly mean SST in SG ranged from 9.4 ± 2.7 °C in February to 24.9 ± 1.4 °C in August and in CD from 7.3 ± 2.3 °C in February to 24.7 ± 1.1 °C in August (Figure 2b). The monthly mean significant wave height ranged from 0.4 ± 0.1 m in July to 1.1 ± 0.5 m in January in SG and from 0.6 ± 0.2 m in July to 1.0 ± 0.2 m in January in CD (Figure 2c). The seasonal mean salinity ranged from 32.49 ± 0.85 PSU in summer to 34.15 ± 0.12 PSU in winter in SG and from 32.12 ± 1.19 PSU in summer to 33.98 ± 0.25 PSU in winter in CD (Figure 3).

**Figure 4:**
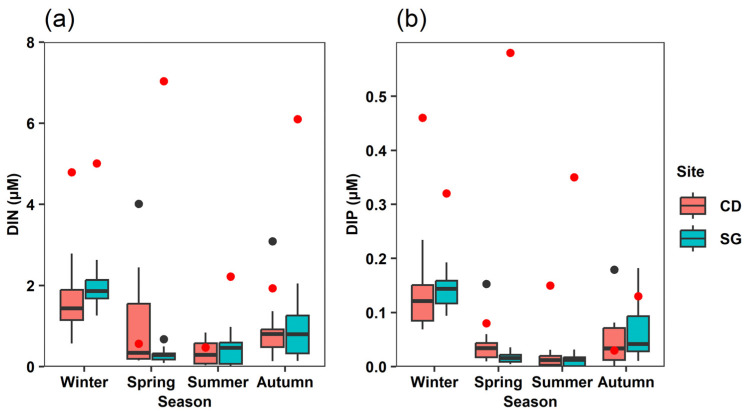
Ten-year (2014–2023) monthly mean nutrient concentrations in Sageunjin (SG) and Chodo (CD). (**a**) Dissolved inorganic nitrogen, DIN; (**b**) dissolved inorganic phosphate, DIP; black dots: outlier data; red dots: field survey data; source: Korea Marine Environment Management Corporation (KOEM).

In SG, the seasonal mean DIN concentration ranged from 0.29 ± 0.31 µM in summer to 1.94 ± 0.40 µM in winter, while in CD it ranged from 0.36 ± 0.31 µM in summer to 1.56 ± 0.63 µM in winter (Figure 4a). The seasonal mean DIP concentration in SG ranged from 0.01 ± 0.01 µM in summer to 0.14 ± 0.03 µM in winter and in CD from 0.01 ± 0.01 µM in summer to 0.13 ± 0.05 µM in winter (Figure 4b).

**Figure 5:**
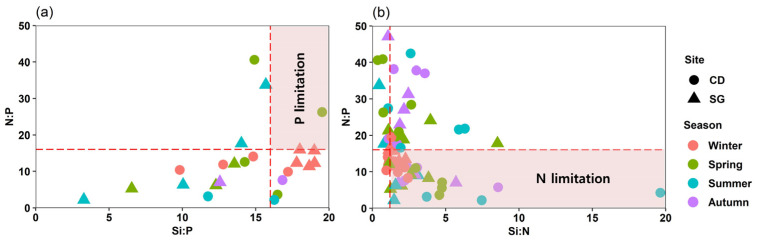
Ten-year (2014–2023) monthly mean elemental ratios in Sageunjin (SG) and Chodo (CD). (**a**) N:P and Si:P ratios; (**b**) N:P and Si:N ratios; source: Korea Marine Environment Management Corporation (KOEM).

The N:P ratio, calculated from seasonal DIN and DIP values, was lower than 16:1 (Redfield ratio) in February (winter) and November (autumn) for both SG and CD (Figure 5).

### 3.2. Seasonal Changes in Biomass of Algal Communities

**Figure 6:**
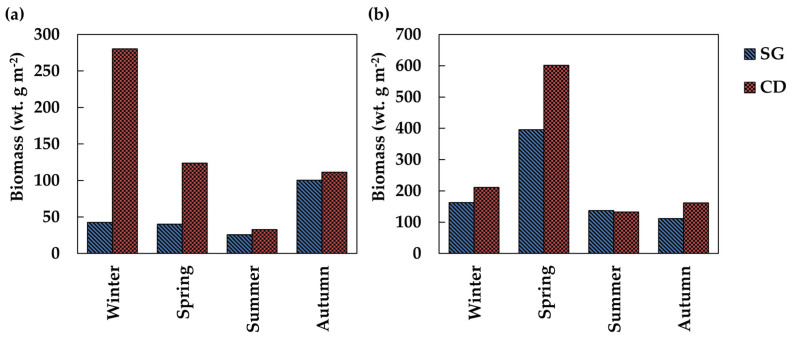
Seasonal changes in biomass in Sageunjin (SG) and Chodo (CD). (**a**) Intertidal zone; (**b**) Subtidal zone.

In the intertidal zone, the seasonal mean biomass of the communities in SG ranged from 25.7 g in summer to 100.4 g in autumn. The CD ranged from 32.7 g in summer to 280.3 g in winter (Figure 6a). In the subtidal zone, the seasonal mean biomass in SG ranged from 111.5 g in autumn to 395.7 g in spring, while in CD it ranged from 132.3 g in summer to 601.3 g in spring (Figure 6b).

### 3.3. Taxonomic Composition of Algal Communities

**Figure 7:**
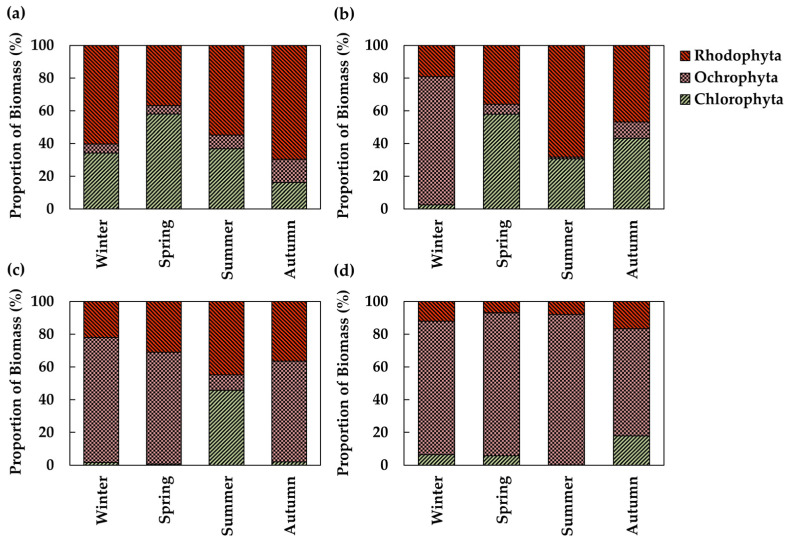
Seasonal changes in the biomass proportion of algal communities by taxonomic group. (**a**) Intertidal zone at Sageunjin (SG); (**b**) intertidal zone at Chodo (CD); (**c**) subtidal zone at Sageunjin (SG); (**d**) subtidal zone at Chodo (CD).

In the intertidal zone of SG, Chlorophyta ranged from 16.2% (16.2 g) in autumn to 58.0% (23.3 g) in spring. Ochrophyta ranged from 5.2% in spring (2.1 g) to 14.1% in autumn (14.1 g). Rhodophyta varied from 36.8% (14.7 g) in spring to 69.7% (70.0 g) in autumn. Rhodophyte generally showed higher proportions, except in spring when green algae dominated (Figure 7a). In the intertidal zone of CD, Chlorophyta ranged from 2.5% (6.9 g) in winter to 57.9% (71.7 g) in spring. Ochrophyta ranged from 1.1% (0,4 g) in summer to 78.4% in winter, with a maximum biomass of 219.9 g. Rhodophyta ranged from 19.1% (53.5 g) in winter to 68.3% (22.3 g) in summer. Ochrophyta were most abundant in winter, while Rhodophyta showed the highest proportion in summer (Figure 7b). In the subtidal zone of SG, Chlorophyta ranged from 0.7% (2.9 g) in spring to 45.7% (62.8 g) in summer. Ochrophyta ranged from 9.5% (13.0 g) in summer to 76.5% (124.3 g) in winter. Rhodophyta ranged from 21.9% (35.7 g) in winter to 44.8% (61.5 g) in summer. Ochrophyta showed the highest proportions in winter, while Chlorophyta and Rhodophyta were more abundant in summer (Figure 7c). In the subtidal zone of CD, Chlorophyta ranged from 0.2% (0.2 g) in summer to 18.0% (29.0 g) in autumn. Ochrophyta ranged from 65.6% (105.8 g) in autumn to 92.0% (121.6 g) in summer. Rhodophyta ranged from 6.8% (41.2 g) in spring to 16.6% (26.8 g) in autumn. Ochrophyta were most dominant in summer, while Chlorophyta and Rhodophyta increased slightly in autumn (Figure 7d).

### 3.4. Functional Group Composition of Algal Communities

**Figure 8:**
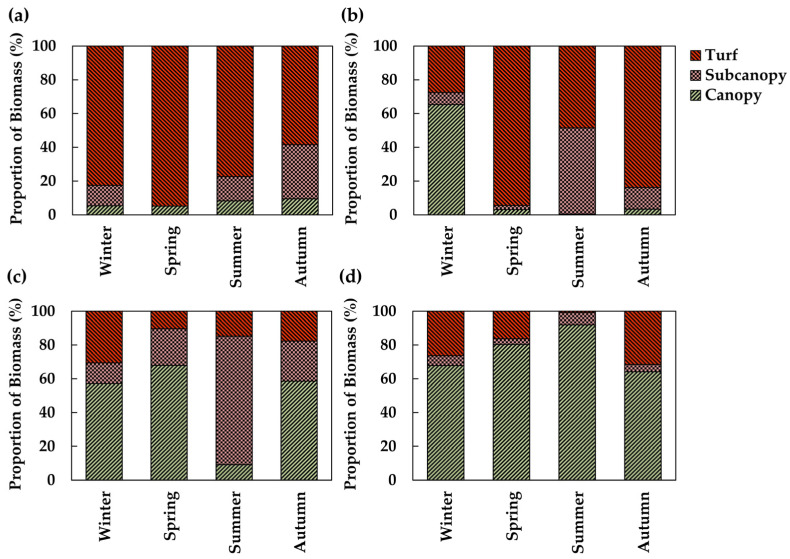
Seasonal changes in the biomass proportion of algal communities by functional group. (**a**) Intertidal zone at Sageunjin (SG); (**b**) intertidal zone at Chodo (CD); (**c**) subtidal zone at Sageunjin (SG); (**d**) subtidal zone at Chodo (CD).

In the intertidal zone of the SG, the biomass proportions by functional groups ranged from 5.1% (2.1 g) in spring to 9.5% (9.6 g) in autumn for canopy species. The proportion of subcanopy species ranged from 0.1% in spring (0.03 g) to 32.0% in autumn (32.1 g). Turf species were dominant, ranging from 58.5% in autumn (58.7 g) to 94.8% in spring (38.0 g) (Figure 8a). In the intertidal zone of CD, canopy species accounted for 0.4% (0.2 g) in summer and increased to 65.3% in winter (183.1 g). Subcanopy species ranged from 2.4% in the spring (3.0 g) to 50.9% in the summer (16.6 g). Turf species contributed 27.7% in the winter (77.6 g) and peaked at 94.4% in the spring (116.9 g) (Figure 8b). In the subtidal zone of SG, canopy species accounted for 9.1% (12.6 g) in summer and increased to 68.0% (269.0 g) in spring. Subcanopy species ranged from 12.4% (20.1 g) in winter to 76.1% (104.5 g) in summer. Turf species ranged from 10.3% (40.9 g) in spring to 30.5% (49.6 g) in winter (Figure 8c). In the subtidal zone of CD, canopy species ranged from 64.2% (103.7 g) in winter to 92.0% (121.6 g) in summer. Subcanopy species varied from 3.4% (7.1 g) in spring to 7.3% (9.6 g) in summer. Turf species contributed 0.8% (1.1 g) in summer and peaked at 31.4% (50.8 g) in autumn (Figure 8d).

### 3.5. Order-Level Group Composition of Algal Communities

**Figure 9:**
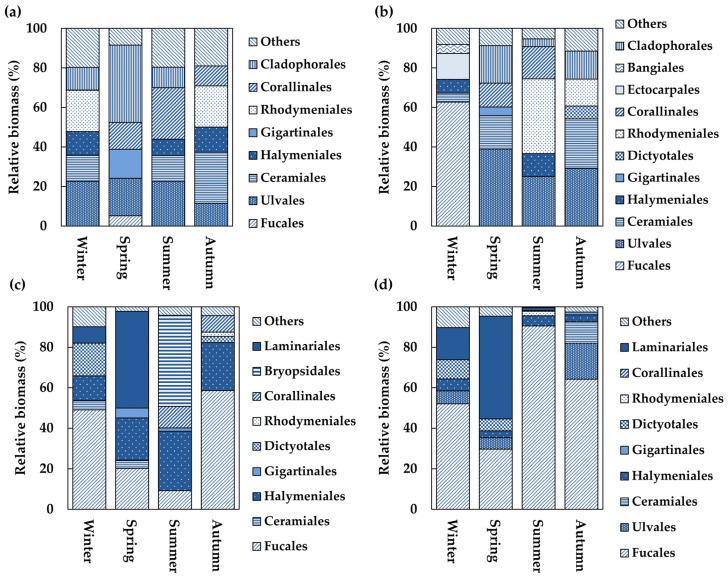
Seasonal changes in the biomass relative proportion of algal communities by order group. (**a**) Intertidal zone at Sageunjin (SG); (**b**) intertidal zone at Chodo (CD); (**c**) subtidal zone at Sageunjin (SG); (**d**) subtidal zone at Chodo (CD).

In the intertidal zone of the SG, the proportion of major Chlorophyta species ranged from 11.5% to 22.6% for Ulvales, with the lowest in autumn and the highest in winter, and from 4.5% to 39.1% for Cladophorales, with the lowest in autumn and the highest in spring. Among the Ochrophyta, Fucales ranged from 5.1% in spring to 9.5% in autumn. The Rhodophyta included Halymeniales, ranging from 0.1% in spring to 12.7% in autumn; Rhodymeniales, ranging from 3.7% in spring to 20.9% in winter and autumn; Ceramiales, ranging from 4.1% in spring to 25.8% in autumn; Gigartinales, ranging from 0% in summer to 14.8% in spring; and Corallinales, ranging from 4.1% in winter to 26.1% in summer (Figure 9a). In the intertidal zone of CD, Ulvales ranged from 2.4% in winter to 38.9% in spring, and Cladophorales ranged from 0% in winter to 19.0% in spring, with a summer value of 4.0%. The Ochrophyta included Fucales, ranging from 0.4% in summer to 62.6% in winter; Ectocarpales, ranging 2.3% in spring and 13.1% in winter; and Dictyotales, which were absent in winter but ranged from 0.3% in spring to 6.6% in autumn. Rhodophyta included Halymeniales, ranging from 0.2% in autumn to 11.6% in summer; Rhodymeniales, with 13.6% in autumn and a peak of 37.9% in summer; Corallinales, ranging from 2.9% in winter and autumn to 16.3% in summer; Ceramiales, ranging from 0.6% in summer to 25.1% in autumn; Gigartinales, ranging from 0.1% in winter to 4.4% in spring; Bangiales, which were absent in summer but ranged from 0.1% in spring to 4.5% in winter (Figure 9b). In the subtidal zone of the SG, Chlorophyta were dominated by Bryopsidales, accounting for 45.0% in summer. Ochrophyta ranged from 0.3% in spring and summer to 16.2% in winter, with Laminariales reaching 47.8% in spring and 8.1% in winter and Fucales ranging from 9.1% in summer to 58.5% in autumn. Rhodophyta included Halymeniales, which ranged from 12.1% in winter to 29.3% in summer; Gigartinales, ranging from 0.6% in autumn to 4.8% in spring; and Corallinales, which were absent in spring but increased from 3.4% in winter to 10.6% in summer (Figure 9c). In the subtidal zone of the CD, Chlorophyta ranged from 0.2% in summer to 17.8% in autumn. Ochrophyta were dominant, accounting for 50.7% of the total, with Fucales accounting for 29.6% in winter and peaking at 90.5% in summer. Some orders of Ochrophyta were absent in summer, whereas minor proportions were recorded in autumn (1.3%) and winter (9.5%). Rhodophyta was present at 3.4%, with Halymeniales reaching 3.9% in both spring and autumn (Figure 9d).

### 3.6. Seasonal Changes of Ecological Indices

**Figure 10:**
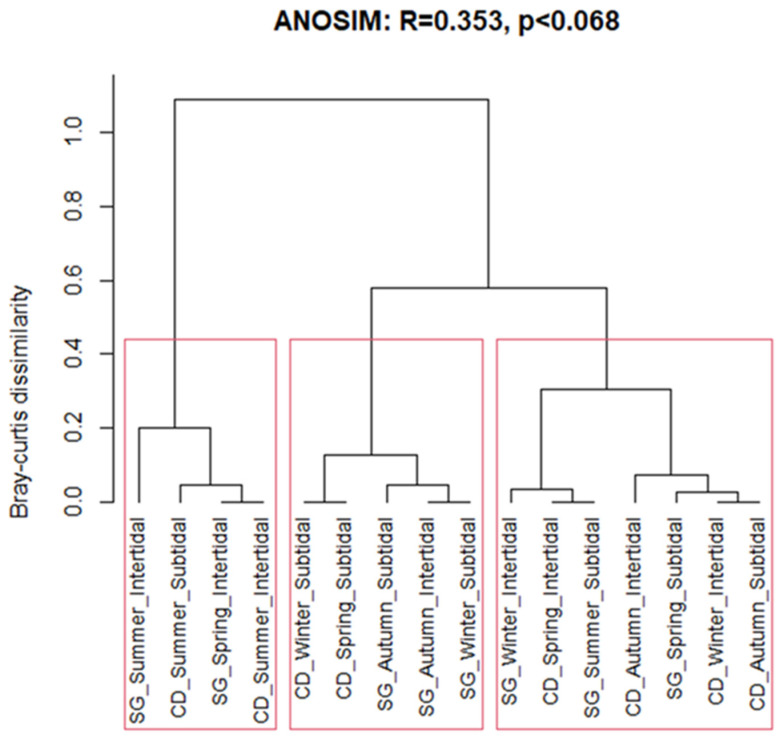
Hierarchical clustering analysis of seasons at Chodo (CD) and Sageunjin (SG).

The EEI-c increased as the proportion of relatively slow-growing, long-lived species increased. In other words, it increased when there were more ESG I species than ESG II species. In the intertidal zone of the SG, EEI-c values ranged from 1.76, indicating a poor ecological status, to 4.27, reflecting a low-to-moderate status in summer. In the intertidal zone of CD, the values ranged from 1.59, indicating a bad status, to 7.99, corresponding to a moderate-to-good status in winter. In the subtidal zone of SG, EEI-c ranged from 2.34 in summer, indicating a bad status, to 8.53 in spring, reflecting a good-to-high status. In the subtidal zone of CD, values ranged from 6.50 in autumn, indicating a moderate-to-good ecological status, to 10 in summer, indicating a high ecological status (Table 2). EQR, like EEI-c, increased as ESG I species were higher relative to ESG II species, with the difference being that it increased as the proportion of Rhodophyta increased or as the proportion of Chlorophyta decreased. In the intertidal zone of SG, the EQR ranged from 0.18 in spring, indicating a bad ecological status, to 0.81 in autumn, indicating a high status. In the intertidal zone of the CD, it ranged from 0.49 to 0.76, corresponding to a moderate-to-good status, with the highest value observed in winter. In the subtidal zone of SG, values ranged from 0.72 in spring to 0.88 in autumn, indicating good-to-high ecological status. In the subtidal zone of the CD, the EQR ranged from 0.55 in summer to 0.76 in both winter and spring, indicating a moderate-to-good status (Table 2). Both species diversity and richness were relatively low during the summer across both study sites. However, the ecological evaluation index (EEI-c) showed comparatively higher values in the summer at the intertidal zone of Sagunjin and the subtidal zone of Goseong. Similarity analysis identified three distinct clusters with more than 60% Bray–Curtis similarity (Table 2). Notably, the subtidal zone of Chodo in winter and spring, the subtidal zone of Sagunjin in winter and fall, and the intertidal zone of Sagunjin in fall exhibited over 80% similarity. Likewise, the intertidal and subtidal zones of Chodo in summer, along with the intertidal zone of Sagunjin in summer and spring, also shared more than 80% similarity (Figure 10).

### 3.7. Key Environmental Factors Influencing Algal Community Structure

**Figure 11:**
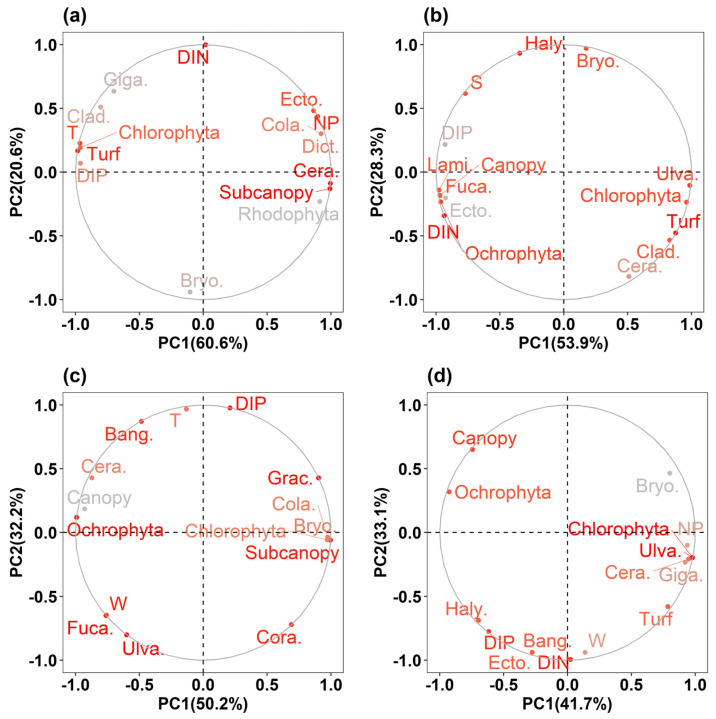
Principal component analysis (PCA) of major environmental and biological factors influencing algal community structure. (**a**) Intertidal zone at Sageunjin (SG); (**b**) intertidal zone at Chodo (CD); (**c**) subtidal zone at Sageunjin (SG); (**d**) subtidal zone at Chodo (CD).

In the PCA results for the changes in the intertidal zone of the SG, PC1, which explained 60.6% of the variance, had high positive loadings for the N:P ratio and subcanopy species. Water temperature, turf species, and Chlorophyta showed relatively negative loadings. Therefore, PC1 explains the changes in community functional groups in environments with low water temperatures and high N:P ratios. PC2 (20.6% explained variance) had high positive loadings for DIN concentration, followed by the remaining Cladophorales. PC2 explained the changes in the community order groups in environments with high DIN concentrations (Figure 11a). In the intertidal zone of the CD, PC1 (53.9% of the explained variance) had high positive loadings for turf species, Chlorophyta, and Ulva. DIN, canopy species, and Ochrophyta had relatively negative loadings. Therefore, PC1 explains the changes in community functional groups in environments with low DIN concentrations. PC2 (28.3% of explained variance) showed positive loadings for salinity, Halymeniales, and Bryopsidale. PC2 explained the changes in the community order groups in environments with high salinity (Figure 11b). In the subtidal zone of the SG, PC1 (50.2% of the explained variance) had high positive loadings for subcanopy species. Canopy species had a relatively negative loading. PC1 explained the changes in community functional groups in environments with low wave heights. PC2 (32.2% of the explained variance) had high positive loadings for water temperature and DIP. Therefore, PC2 explained the changes in the community order groups in environments with high water temperatures and DIP concentrations (Figure 11c). In the CD subtidal zone, PC1 (41.7% explained variance) had high positive loadings for canopy species. Turf species had a negative loading. PC1 explains the competition between the canopy and turf species. PC2 (33.1% explained variance) had negative loadings for the DIN and DIP concentrations. PC2 explained the changes in the community order groups in environments with high DIN and DIP concentrations (Figure 11d).

## 4. Discussion

Increased anthropogenic activity in coastal zones—including shoreline development, port operations, and wastewater discharge—can elevate nutrient inputs into adjacent marine waters, potentially leading to eutrophication under extreme conditions [52,53,54,55]. The study sites investigated in this research are located adjacent to coastal settlements, making them potentially susceptible to human-induced impacts. However, ten-year averages of salinity measurements exhibited low variability, and concentrations of dissolved nutrients were notably lower than those previously reported for the eastern coastal waters of Korea [56,57]. These findings suggest that the influence of anthropogenic nutrient enrichment was not persistent at these locations, and that biological responses were more likely driven by natural environmental factors. Another possible anthropogenic factor is the harvesting and overexploitation of macroalgae, which can affect biomass dynamics within benthic algal communities. Nevertheless, our findings showed that the biomass of commercially important Laminariales species followed previously established seasonal patterns [58,59], implying that the influence of harvesting pressure on community structure was limited during the study period.

The algal community in SG, located south of the SPF zone and more influenced by cold-water masses, contained a high proportion of species with broad environmental tolerance. In contrast, the CD community, located north of the SG and influenced by cold-water masses, was dominated by species with different environmental preferences. In SG and CD, taxa were extremely dominant in the winter intertidal and summer subtidal zones. Furthermore, the main factors affecting seasonal changes differed between regions, with water temperature, followed by DIN concentration, and wave height being the main factors affecting seasonal changes in the community at SG. At CD, the DIN and DIP concentrations were the main factors, followed by wave height and salinity. In both regions, the contributions of the measured variables were relatively low compared to those of the other environmental factors.

Localized environmental differences among regions can lead to distinct algal community structures [60]. For example, canopy species tend to dominate in wave-exposed areas, whereas turf species are more prevalent in sheltered environments [9]. However, our study revealed an unexpected pattern: despite SG experiencing higher average wave heights than CD during winter, the relative abundance of canopy species was greater at CD. This discrepancy may be attributed to differing environmental drivers across the sites—while wave exposure appeared to be the primary factor shaping the winter assemblage at SG, nutrient concentrations likely exerted a stronger influence on the algal community structure at CD during the same period.

Bryopsidales were dominant in the subtidal zone of the SG during the summer. However, their abundance tends to increase in environments where canopy species such as Fucales decline during summer and autumn, as these species can inhibit the growth of Bryopsidales. Bryopsidales also tolerate high water temperatures and low salinity and exhibit a broad geographic distribution [51,61,62,63].

Halymeniales occur year-round in the SG and CD subtidal zones, and some Halymeniales species exhibit a wide range of water temperatures and salinities in which they can survive and grow [64,65]. For example, Halymeniales can grow in a water temperature range of 5–25 °C or salinity range of 20–38 PSU [64] and is considered an invasive species that has been introduced and established from the native coast of Japan to the Mediterranean Sea, the Atlantic coasts of Europe and North America, and even the coasts of Australia and New Zealand [66,67,68,69]. Therefore, Halymeniales can be distinguished visually even when their life-history stages change and are highly tolerant to changes in water temperature and salinity. Therefore, they can be found year-round in the SG and CD subtidal zones.

In CD, the proportion of Fucales increased in the intertidal and subtidal zones during winter and summer, respectively. Long-living and slow-growing seaweeds, such as Fucales, generally exhibit lower nutrient uptake rates than short-living, fast-growing species, such as Cladophorales [70]. Although Fucales grow slowly and have relatively low uptake rates, they can store sufficient nutrients in their tissues to endure periods of low nutrient availability [71]. In addition, there were species belonging to the order Fucales that can grow in a relatively higher range of water temperatures than Laminariales, thriving primarily in winter when water temperatures are low in the tropics and in spring or summer in temperate regions [72,73]. Thus, in the CD subtidal zone, Fucales would have dominated in summer, when the water temperatures were relatively high. In contrast, in the CD intertidal zones, the proportion was only high in winter when nutrient concentrations were high. Algae formed horizontal distribution zones based on their tolerance to desiccation and wave-induced stresses in the intertidal zones of both sites [10]. In particular, the dominance of Fucales may decrease towards the upper intertidal zone because they have morphological and physiological traits adapted to aquatic environments rather than those exposed to the atmosphere [10,74,75,76].

Laminariales were common in the subtidal zones of both SG and CD, exhibiting distinct seasonality. They typically emerged from winter to early spring, peaked in spring, and declined in summer. This seasonal pattern reflects a heteromorphic life cycle of microscopic filamentous gametophytes. The water-temperature tolerances of these stages differ, with gametophytes surviving at higher water temperatures than sporophytes [58,59]. For example, Laminariales (e.g., *U. pinnatifida*) spores can grow at 5–27 °C, while thallus bleaching and mortality occur above 28 °C [59]. Hermaphroditic gametophytes grow between 10 °C and 28 °C and die above 29 °C, indicating greater thermal tolerance than sporophytes [58,59]. These morphological and thermal differences between life stages may serve as survival strategies during warm periods, as Laminariales generally prefer cold-water environments. In species such as *Saccharina* spp., sporophytes decay under high water temperatures, whereas gametophytes released during summer persist, allowing the population to endure unfavorable conditions [77]. Although there were differences in the seasonal changes in N:P ratios in SG and CD, the time of year below 16 was the same in February (winter) and November (autumn), suggesting that the N:P ratio is likely a common major factor influencing seasonal changes in the algal community structure in SG and CD in the subtidal zone. DIN and DIP are the primary nutrients limiting algal growth [78,79]. Furthermore, algae have mean C:N:P elemental ratios of 550:30:1 [80] or 800:49:1 [81], which are higher than the 106:16:1 (the Redfield ratio) for phytoplankton. Therefore, most algae require higher DIN concentrations than do phytoplankton.

## 5. Conclusions

The aim of this study was to investigate seasonal changes in the algal community structure along the ECK, an SPF zone, and identify the key environmental factors influencing this structure. The dominant algal species differed between SG and CD, with wave-resistant species commonly dominating the intertidal zones at both sites. However, a shift toward the dominance of Fucales occurred in the intertidal zone during winter. Turf species were positively correlated with water temperature in SG, whereas canopy species were positively correlated with nutrient concentration in CD. In the subtidal zones of both the SG and CD, Laminariales dominated during winter and spring. As Laminariales declined in summer, Bryopsidales became dominant in the SG, whereas Fucales replaced them in the CD. In summer, Bryopsidales in SG were positively correlated with N:P ratio, whereas those in CD were positively correlated with water temperature. These findings suggested that in dynamic environments, such as the SPF zone, even geographically proximate sites may exhibit distinct seasonal patterns in algal community composition, likely driven by site-specific environmental factors.

## Figures and Tables

**Table 1:** Classification of algae based on ecological structure and thallus morphology [42,43,44].

Functional Group	Thallus Size	Growth Form
Canopy species	>1 m	Erect
Subcanopy species	0.05–1 m	Erect
Turf species	<5 cm	Erect or prostrate
Encrusting species	Not erect	Covering the substratum

**Table 2:** Seasonal ecological evaluation index (EEI-c) and ecological quality ratio (EQR) for algal communities at Sageunjin (SG) and Chodo (CD).

	Site	Season	Richness	Diversity	EEI-c (0–10) ^1^	EQR (0–1) ^2^
Intertidal zone	SG	Winter	19	2.03	1.76	0.53
		Spring	15	1.85	3.84	0.18
		Summer	11	1.57	4.27	0.36
		Autumn	30	2.73	2.95	0.81
	CD	Winter	23	2.16	7.99	0.76
		Spring	18	1.93	2.23	0.49
		Summer	15	1.81	2.12	0.55
		Autumn	21	2.12	1.59	0.59
Subtidal zone	SG	Winter	30	2.69	7.61	0.80
		Spring	24	2.20	8.53	0.72
		Summer	18	1.99	2.34	0.75
		Autumn	28	2.29	7.47	0.88
	CD	Winter	26	2.25	6.62	0.76
		Spring	26	2.26	8.92	0.76
		Summer	14	1.74	10.00	0.55
		Autumn	23	2.20	6.50	0.67

^1^ Ecological status classes based on each index; EEI-c: Bad (2), Low (4); Moderate (6); Good (8); High (10); ^2^ EQR: Bad (0.0–0.2); Poor (0.2–0.4); Moderate (0.4–0.6); Good (0.6–0.8); High (0.8–1.0).

## Data Availability

Dataset available on request from the authors.

## Abbreviations

The following abbreviations are used in this manuscript:

SPF	Subpolar front
SST	Sea surface temperature
ESG	Ecological status group
EEI-c	Ecological evaluation index
EQR	Ecological quality ratio
PCA	Principal component analysis
PC	Principal component
DIN	Dissolved inorganic nitrogen
DIP	Dissolved inorganic phosphate
CD	Chodo
SG	Sageunjin
ECK	Eastern coast of Korea
KMA	Korea Meteorological Administration
KOEM	Korea Marine Environment Management
